# Curcumin delivery system based on biodegradable polyhydroxybuterate Chitosan copolymer and Cobalt oxide nanoparticles against colorectal cancer

**DOI:** 10.1038/s41598-025-34587-y

**Published:** 2026-03-09

**Authors:** Nehal Salahuddin, Mohamed Gaber, Maie Mousa, Mona Elfiky

**Affiliations:** Chemistry Department, Faculty of Science, Tanta University, Tanta, 31527 Egypt

**Keywords:** Curcumin, Chitosan, Polyhydroxybutyrate, Cobalt oxides, Good health, Biotechnology, Cancer, Chemistry, Materials science, Nanoscience and technology

## Abstract

In this study, a biodegradable nanocomposite composed of polyhydroxybutyrate-co-chitosan and cobalt oxide (PHB-co-CS/Co_3_O_4_) was developed for targeted and sustained in-vitro release of curcumin (CUR). The nanocomposite was prepared by varying the concentrations of Co_3_O_4_ nanoparticles (NPs) and using chitosan with different molecular weights (LCS, HCS) to optimize the release profile. The PHB-co-CS copolymer was synthesized through a coupling reaction between PHB-diol and the terminal isocyanate groups of chitosan, using stannous octanoate as a catalyst. PHB-diol was obtained via transesterification of PHB with ethylene glycol. Amine group in CS was protected by phthalic anhydrides followed by reaction with hexamethylene diisocyanato. Co_3_O_4_ NPs were synthesized by reacting cobalt acetate with sodium carbonate in ethylene glycol. CUR was first loaded onto the Co_3_O_4_ NPs and then dispersed within the PHB-co-CS copolymer matrix. Structural and chemical characterization was performed using X-ray diffraction (XRD) and Fourier transform infrared (FTIR) spectroscopy. In-vitro release studies were conducted in buffer solutions at pH 5.4 and 7.4, showing enhanced release in acidic conditions, which supports tumor-specific targeting. Formulations with lower molecular weight chitosan released curcumin more rapidly. Among the tested systems, the PHB-co-LCS/10%CUR@Co_3_O_4_ nanocomposite exhibited the strongest anticancer effect, with IC_50_ of 29.1 µg/mL against HCT-116 colorectal cancer cells. These results highlight the promise of combining green materials and sustainable approaches to develop effective, targeted drug delivery platforms for cancer treatment.

**Supplementary Information:**

The online version contains supplementary material available at 10.1038/s41598-025-34587-y.

## Introduction

Although clinical cancer treatments have advanced considerably, cancer remains a major global health challenge, causing millions of deaths each year. Despite the development of various therapeutic approaches, the incidence and mortality rates of many cancers have not significantly declined over the past three decades. This emphasizes the urgent need to deepen our understanding of the molecular and cellular mechanisms involved in cancer initiation and progression, which is crucial for improving prevention and treatment^1^. As a result, there is increasing interest in discovering safer and more effective alternatives to traditional anticancer drugs. Among these, natural compounds from plant and animal sources have attracted widespread attention due to their promising therapeutic properties and relatively low toxicity^2^.

Curcumin (CUR), a natural polyphenol identified chemically as [1,7-bis(4-hydroxy-3-methoxyphenyl)-1,6-heptadiene-3,5-dione], is extracted from the turmeric plant (*Curcuma longa*), a member of the Zingiberaceae family. Traditionally used in Ayurvedic medicine, CUR is recognized for its low toxicity and diverse pharmacological activities, including anti-inflammatory, antioxidant, antiseptic, and analgesic effects. Recent studies have highlighted its anticancer potential through regulation of key biological pathways such as oncogene expression, cell cycle control, apoptosis, and metastasis^3,4^. However, its clinical application is limited by poor bio-availability and rapid metabolism^5^. To address these challenges, CUR has been incorporated into polymer-based nanocarriers, which improve its stability and therapeutic efficacy. These systems, particularly when applied in combination therapies, have shown encouraging results in enhancing cancer treatment outcomes^6^. In addition, Curcumin-loaded mesoporous silica nanoparticles (MSNs) enable targeted delivery to specific cells and tissues, enhancing therapeutic efficacy^7^.

Polyhydroxybutyrate (PHB) is a bio-based polyester of considerable interest due to its complete biodegradability and microbial origin, as it is naturally produced by bacteria as intracellular inclusion bodies^8,9^. PHB is also biocompatible, eliciting minimal foreign-body response when implanted. PHB offers a hydrophobic, crystalline structure that enhances NP rigidity, improves long-term stability, and slows water penetration into the matrix. These attributes enable more efficient drug entrapment and facilitate a prolonged and controlled release of Perindopril Erbumine^10^. Therefore, the choice of PHB extends well beyond its biodegradability, representing a deliberate design strategy to refine the mechanical and release properties of the chitosan-based system^11^. To address this limitation, PHB is commonly copolymerized with chitosan (CS), which enhances its biodegradability, mechanical strength, and structural stability^12^.

Chitosan is a biodegradable, cationic polysaccharide naturally derived from the exoskeletons of marine crustaceans. It is highly functionalizable and approved by the U.S. Food and Drug Administration for biomedical use^13^. Thanks to its bioactivity, low toxicity, mucoadhesive properties, and structural versatility, chitosan is considered a promising candidate for targeted drug delivery in both diagnostic and therapeutic contexts^14,15^. In the body, chitosan is enzymatically degraded by chitosanases and lysozymes into absorbable oligosaccharides and monosaccharides. Its degradation rate and solubility are influenced by molecular weight, with lower molecular weight forms degrading more rapidly and exhibiting higher solubility^16^. Incorporating chitosan into PHB enhances its moisture absorption, lowers the contact angle, and modifies its mechanical behavior. The properties of the composite can be tailored, By adjusting the amount of chitosan, making it suitable for various biomedical applications^17^. Chitosan–itaconic acid copolymers blended with starch and carbon black revealed improvement in thermal stability and biodegradation, and the optimum percentage of B15 (1% grafted copolymer, 5% starch) blend was estimated to introduce the highest performance^18^. Moreover, chitosan-coated cobalt oxide (NPs) have demonstrated improved solubility in acidic conditions, enhancing cobalt ion release and exhibiting better compatibility with normal cells^19^. Notably, effective localized cancer therapy has been achieved using systems such as doxorubicin-loaded titanium oxide nanofibers, electrospun chitosan, and cobalt ferrite, with drug release enhancement under acidic conditions and magnetic fields^20^.

Cobalt oxide (Co₃O₄) is a promising material for biomedical applications due to its distinct physical and chemical properties, such as curie temperature, coercivity, and anisotropy constant, along with its straightforward synthesis^21,22^. As an antiferromagnetic, p-type semiconductor with direct optical band gaps of 1.48 and 2.19 eV, Co₃O₄ has been extensively investigated for use in cancer therapy^23^, magnetic resonance imaging (MRI)^24^, and targeted drug delivery^25^. Co₃O₄ NPs have demonstrated anticancer activity by inhibiting the proliferation of K562 leukemia cells through both intrinsic and extrinsic apoptotic pathways. and exhibit antimicrobial properties^26^. These NPs may also induce autolysosome accumulation and interfere with lysosomal function by disrupting intracellular degradation processes and reducing ATP levels^27^.

This study developed a PHB-co-CS/Co₃O₄ nanocomposite for the encapsulation and controlled release of CUR in colorectal cancer therapy. The copolymer was synthesized via a coupling reaction between the terminal hydroxyl groups of PHB-diol and the isocyanate functionalized chitosan, using chitosan of varying molecular weights. Co_3_O₄ NPs with ferromagnetic behavior can be used in hyperthermia to generate heat for cancer treatment by applying alternating magnetic field. The effect of incorporating Co₃O₄ NPs on CUR release was examined, along with drug loading efficiency at different CUR concentrations. Release kinetics were evaluated at pH 5.4 and 7.4 to mimic tumor and physiological environments, respectively. Cytotoxicity of the CUR-loaded nanocomposites was assessed in HCT-116 colorectal cancer cells and compared to the activity of Co₃O₄ NPs alone.

## Materials and experimental

### Materials

Purified poly(3-hydroxybutyrate) (PHB; M_v_ = 425.51 × 10³ g/mol) was supplied by PHB Industrial S.A. (Brazil), supported by Prof. Emo Chiellini, University of Pisa, Italy. Cobalt acetate tetrahydrate [Co(CH₃COO)₂·4 H₂O] was obtained from Merck (Darmstadt, Germany). Two grades of chitosan were employed: high molecular weight (HCS, 100,000–300,000 g/mol, ≥ 85% deacetylated; Acros Organics) and low molecular weight (LCS, 50–90 kDa, 98.08% deacetylated). Ethylene glycol (Reagent Plus, ≥ 99.0%), phthalic anhydride (Across, India), sodium carbonate (Na₂CO₃) (Reagent Plus, ≥ 99.0%), and various solvents (methanol, chloroform, 1,2-dichloromethane, diethyl ether, ethanol, acetone, dimethylformamide (DMF) anhydrous, and hydrazine monohydrate) were used as received. Hexamethylene diisocyanate (HDI; ≥98.0% purity by GC; M_v_ = 186.19 g/mol; C₈H₁₂N₂O₂) was sourced from Germany. CUR, the model drug, was purchased from Sigma-Aldrich and used without further purification. Moreover, Colorectal carcinoma Colon cancer (HCT-116) cell line was obtained from ATCC via holding company for biological products and vaccines (VACSERA), Cairo, Egypt.

### Preparation of PHB-Co-CS copolymer

#### Phthaloylation of Chitosan (PHCS)

Phthaloyl chitosan (PHCS) was synthesized by reacting low molecular weight CS (LCS; 6.2 mmol, 1 g) with an excess of phthalic anhydride (18.23 mmol, 2.7 g) in DMF containing 5% distilled water (DW) (v/v). The reaction was carried out under a nitrogen atmosphere at 120 °C for 8 h. After cooling to room temperature, the mixture was poured into ice-cold water to precipitate the product, which was then filtered, washed with 150 mL of methanol to remove residual impurities, and dried under vacuum. The yield of purified PHLCS was 70.27%. The same procedure was applied using high molecular weight CS (HCS) to obtain PHHCS, with a yield of 72.05%^28^.

#### Preparation of PHB-diol

Polyhydroxybutyrate-diol (PHB-diol) was synthesized by dissolving 2.5 g (29.069 mmol) of PHB in 25 mL of chloroform. Anhydrous p-toluenesulfonic acid (0.83 g) and ethylene glycol (8.3 mL, 133.72 mmol) were added dropwise to the solution. The reaction mixture was refluxed under a nitrogen atmosphere for 6 h. Upon completion, the mixture was cooled to room temperature and poured into stirred distilled water to precipitate the product. The solid was collected by filtration, washed repeatedly with distilled water, and dried at 60 °C to a constant weight. The final yield of PHB-diol was 3.4 g^29^.

#### Preparation of PHLCS-isocyanate

(PHLCS; 0.0010 mol, 0.3092 g) was dissolved in 10 mL of DMF and reacted with (HDI; 0.0026 mol, 0.44 g) in the presence of stannous octanoate (0.05 g) as a catalyst. The mixture was stirred under a nitrogen atmosphere at 70 °C for 15 h. After completion, the reaction was cooled to room temperature. The same procedure was applied to high molecular weight PHCS to synthesize PHHCS-isocyanate.

#### Synthesis of PHB-co-PHLCS copolymers

PHB-co-PHLCS copolymers were synthesized via a coupling reaction between the terminal hydroxyl groups of PHB-diol and the isocyanate groups of PHLCS-isocyanate in a 1:1 molar ratio. PHB-diol (1.8 g) was dissolved in 20 mL of 1,2-dichloromethane and gradually added to the pre-prepared PHLCS-isocyanate solution at 75 °C under a nitrogen atmosphere. Upon completion, the mixture was cooled to 28 °C, and diethyl ether was added to precipitate the product. The resulting solid was filtered, washed three times with distilled water, and dried under vacuum at 60 °C to a constant weight. The final yield of PHB-co-PHLCS copolymer was 79.41% (2.7 g). The same procedure was used with PHHCS to synthesize PHB-co-PHHCS copolymer, yielding 77.6% (2.64 g).

#### Deprotection of phthaloyl groups to obtain PHB-co-LCS copolymers

**Scheme 1:**
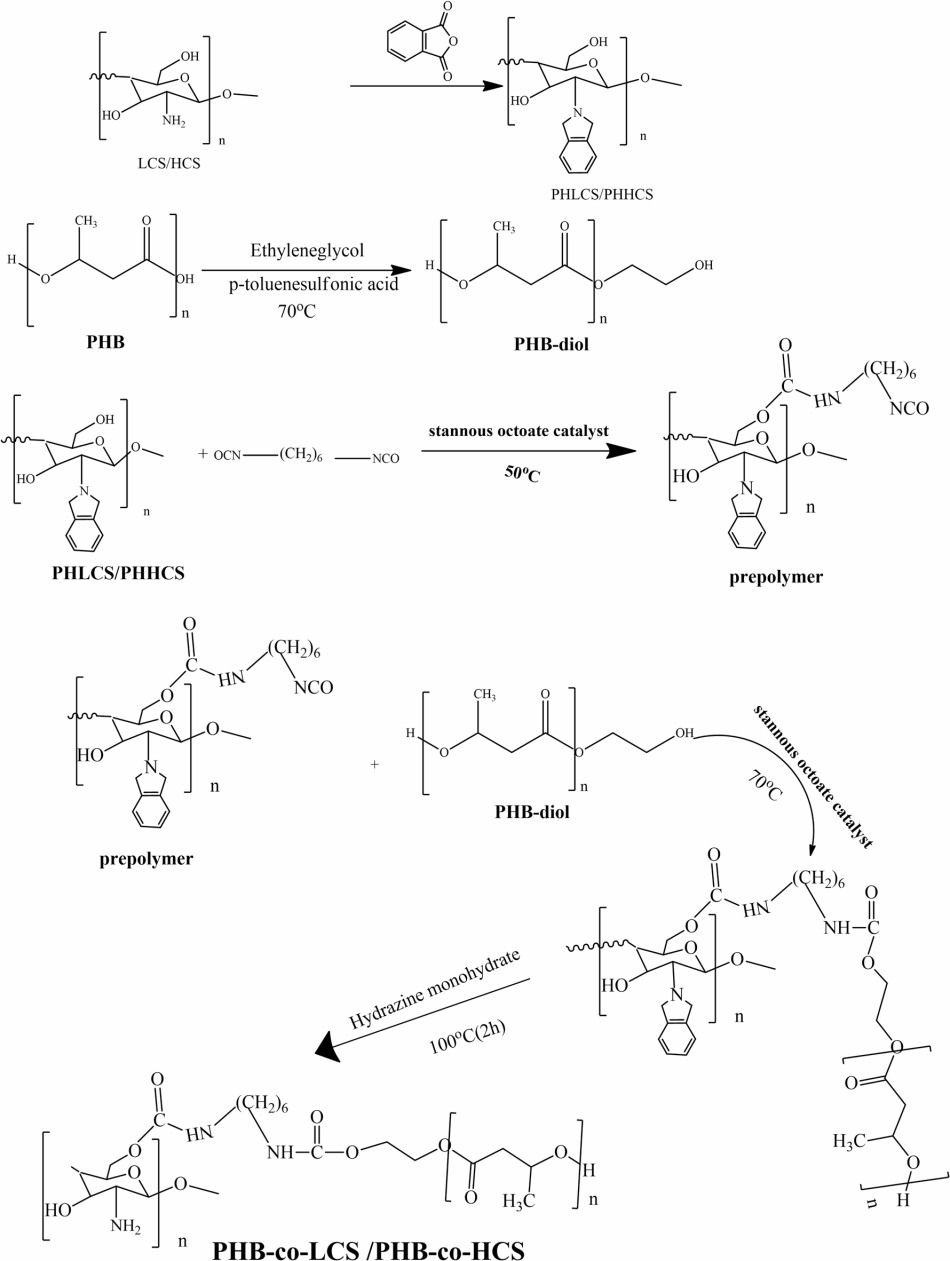
Preparation of PHB-co-LCS /PHB-co-HCS copolymer.

Phthaloyl-protected PHB-co-PHLCS copolymers (1 g) were deprotected by treatment with 2 mL of hydrazine monohydrate at 100 °C for 2 h. After cooling, the product was thoroughly washed with water and ethanol, then dried under vacuum. The final PHB-co-LCS copolymer was obtained with a yield of 58%. An identical procedure was applied to PHB-co-PHHCS copolymers, yielding 57% of PHB-co-HCS (Scheme 1).

### Synthesis of Cobalt oxide NPs

Co₃O₄ NPs were synthesized by dissolving cobalt acetate tetrahydrate (4.98 g) in 60 mL of ethylene glycol. The solution was heated to 160 °C, followed by the addition of 200 mL of a 0.2 M Na_2_CO_3_ aqueous solution using a syringe pump at a controlled flow rate of 1.11 mL/min. The reaction mixture was aged at 160 °C for 1 h. The resulting precipitate was filtered, thoroughly washed with ethanol and distilled water, and dried at 50 °C for 12 h. Finally, the precursor was calcined at 450 °C for 4 h to obtain Co₃O₄ NPs^30^.

### Preparation of PHB-co-LCS-Co_3_O_4_ nanocomposites

PHB-co-LCS (0.95 g) was suspended in chloroform and stirred at 25 °C for 1 h. Cobalt oxide (Co_3_O_4_; 0.05 g) was then added, and the mixture was continuously stirred for 48 h. Following this, the suspension was sonicated for 1 h. The resulting product was precipitated by the addition of excess ethanol, filtered, and dried under vacuum to yield PHB-co-LCS/5%Co_3_O_4_ nanocomposites (NCs). The same procedure was carried out using 0.95 g PHB-co-HCS, 0.05 g Co_3_O_4_, 0.90 g PHB -co-LCS, 0.10 g Co_3_O_4_ and 0.90 g PHB-co-HCS, 0.10 g Co_3_O_4_ to obtain PHB-co-HCS/5%Co_3_O_4_, PHB-co-LCS/10%Co_3_O_4_, and PHB-co-HCS/10%Co_3_O_4_ nanocomposites.

### Curcumin loading onto Co₃O₄ NPs

To load CUR onto cobalt oxide nanoparticles, 0.1 g of Co₃O₄ NPs was added to 100 mL of a CUR solution (dissolving in methanol) (14 ppm) and stirred continuously. After 18 h, the suspension was centrifuged to separate the nanoparticles. The absorbance of the supernatant was then measured at λₘₐₓ = 425 nm using a UV–visible spectrophotometer to determine the concentration of unbound (residual) CUR. The amount of CUR loaded onto the Co₃O₄ nanoparticles was calculated by subtracting the residual drug concentration from the initial concentration.

### Dispersion of Co_3_O_4_ nanoparticles loaded CUR by PHB-co-CS

To prepare the drug-loaded nanocomposites, 0.95 g of PHB-co-LCS was suspended in chloroform, followed by the addition of 0.05 g of CUR@Co_3_O_4_ NPs. The mixture was stirred for 48 h and subsequently sonicated for 1 h to ensure uniform dispersion. The resulting suspension was then precipitated by the addition of excess ethanol, filtered, and dried under vacuum to obtain PHB-co-LCS/5%CUR@Co₃O₄ nanocomposites. The same procedure was carried out using (0.95 g PHB-co-HCS and 0.05 g CUR@Co₃O₄), (0.90 g PHB-co-LCS and 0.10 g CUR@Co₃O₄), and (0.90 g PHB-co-HCS and 0.10 g CUR@Co₃O₄) to obtain PHB-co-HCS/5%CUR@Co₃O₄ nanocomposites, PHB-co-LCS/10%CUR@Co₃O₄ nanocomposites, and PHB-co-HCS/10%CUR@Co₃O₄ nanocomposites, respectively.

### In-vitro drug release study

To assess the release profile of CUR, 100 mg of CUR-loaded nanoparticles was suspended in 20 mL of phosphate-buffered saline (PBS) at pH 5.4 and pH 7.4, representing acidic and physiological conditions, respectively. The suspensions were incubated under controlled conditions, and at predetermined time intervals, aliquots were withdrawn and centrifuged for 10 min to separate the nanoparticles. The concentration of CUR released into the supernatant was quantified using a UV–visible spectrophotometer at 425 nm. The drug release percentage was calculated using the following Eq. (1):

1 $$\:Release\:\% = \frac{{{\mathrm{mass}}\:{\mathrm{of}}\:{\mathrm{drug}}\:{\mathrm{in}}\:{\mathrm{solution}}\:\left( {\mathrm{g}} \right)}}{{{\mathrm{mass}}\:{\mathrm{of}}\:{\mathrm{drug}}\:{\mathrm{loaded}}\:{\mathrm{onto}}\:{\mathrm{nanocomposite}}\:\left( {\mathrm{g}} \right)}} \times 100$$

### Determination of antitumor activity (MTT Assay)

The antitumor activity of nanocomposite formulations was assessed using the MTT assay against HCT-116 human colorectal cancer cells. This colorimetric assay evaluates cell metabolic activity by the enzymatic reduction of yellow tetrazolium bromide (MTT) to insoluble purple formazan via mitochondrial succinate dehydrogenase in viable cells. HCT-116 cells were cultured in RPMI-1640 medium supplemented with 10% fetal bovine serum (FBS), 100 U/mL penicillin, and 100 µg/mL streptomycin, maintained at 37 °C in a humidified atmosphere with 5% CO_2_. Cells were seeded in 96-well plates at a density of 1.0 × 10^4^ cells/well and incubated for 48 h, followed by treatment with varying concentrations of the nanocomposite formulations and further incubation for an additional 48 h. Subsequently, 20 µL of MTT solution (5 mg/mL) was added to each well and incubated for 4 hours. To dissolve the formed formazan crystals, 100 µL of dimethyl sulfoxide (DMSO) was added. Absorbance was recorded at 570 nm using a microplate reader (EXL 800, USA), and relative cell viability was calculated by dividing A₅₇₀ of treated samples by that of untreated controls^31^.

### Characterization

Fourier transform infrared spectroscopy (FTIR) was conducted using a Shimadzu FTIR-8101 A spectrometer over the range of 4000–400 cm⁻¹ with a resolution of 4 cm⁻¹. Samples were prepared as potassium bromide (KBr) pellets and subjected to 64 scans. FTIR analysis was performed to identify functional groups and assess molecular interactions within the synthesized materials. X-ray diffraction (XRD) analysis was carried out using a Philips PW1710 diffractometer with Cu-Kα radiation (λ = 1.54060 Å) to determine the crystalline structure and degree of crystallinity. Scanning was performed over a 2θ range of 2°–80° at a rate of 0.02°/min. High-resolution transmission electron microscopy (HR-TEM) was used to examine nanoparticle morphology and size, utilizing a JEM-2100 (JEOL) instrument. The concentration of curcumin was quantified using UV–Visible spectrophotometry (UV-18000 double beam spectrophotometer) across a wavelength range of 200–600 nm. All measurements were performed in triplicate, and the average values were used for analysis.

## Results and discussion

**Fig. 1:**
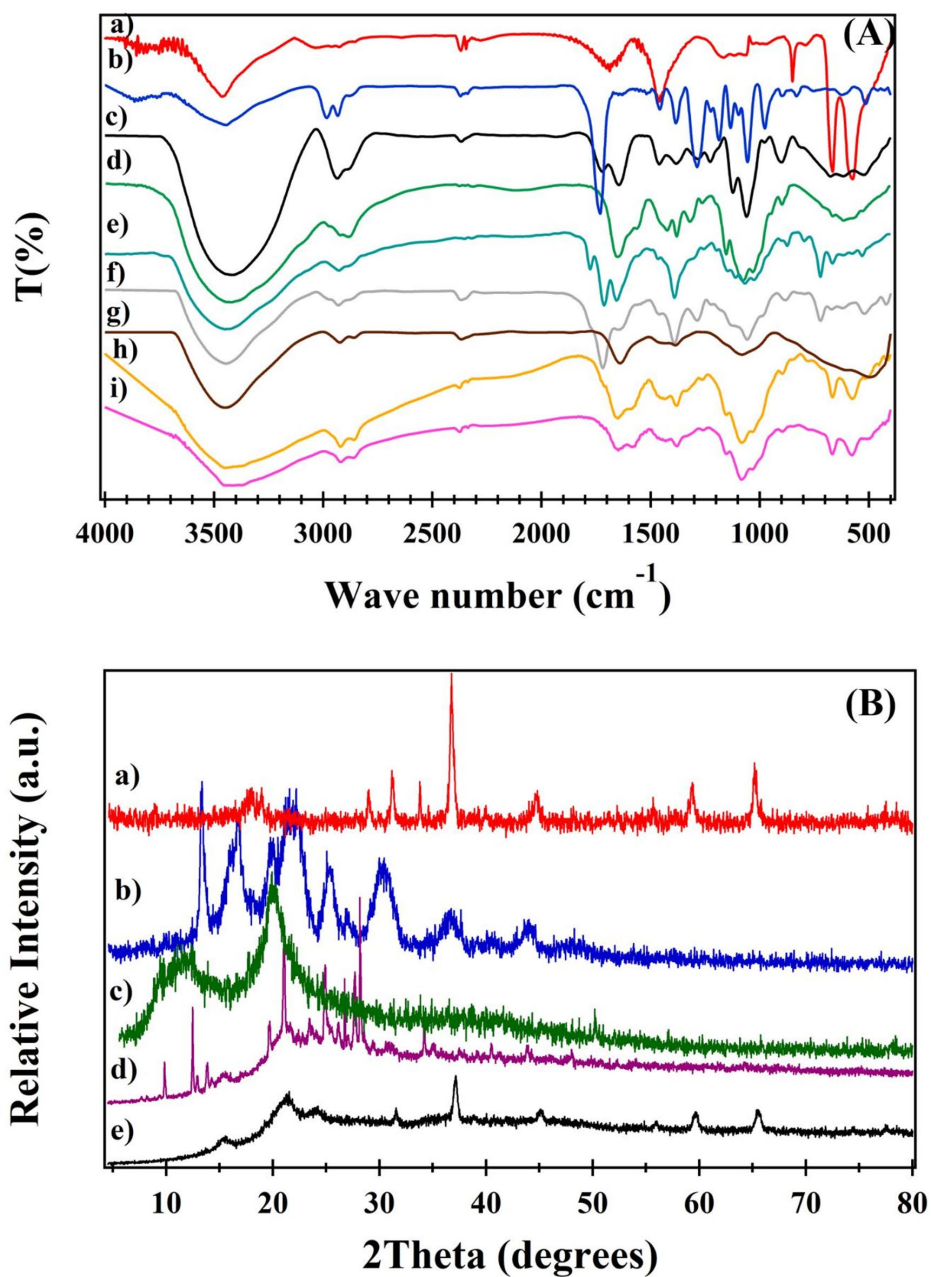
(**A**) FTIR spectra of (a) Co_3_O_4_, (b) PHB, (c) PHB-diol, (d) CS, (e) PHHCS, (f) PHB-Co-PHHCS, (g) PHB-co-HCS, (h) PHB-co-HCS/5%Co_3_O_4_, and (i) PHB-co-HCS/10% nanocomposites and (**B**) XRD pattern of (a) Co_3_O_4_ nanoparticles, (b) PHB, (c) HCS, (d) PHB-co-HCS copolymer and (e) PHB-co-HCS/10%Co_3_O_4_ nanocomposites.

Figure 1A presents the FTIR spectra of Co₃O₄, PHB, PHB-diol, chitosan (CS), PHB-co-PHHCS copolymers, PHB-co-HCS, and the corresponding nanocomposites (PHB-co-HCS/5%Co₃O₄ and PHB-co-HCS/10%Co₃O₄). The Co₃O₄ spectrum (Fig. 1A_a_) displays characteristic bands at 3456.9 and 1638 cm^-1^, attributed to adsorbed water molecules. Sharp absorption peaks at 676 and 575 cm⁻¹ correspond to the stretching vibrations of Co²⁺–O and Co³⁺–O bonds, respectively, consistent with spinel-type Co₃O₄ ^32,33^. PHB (Fig. 1A_b_) shows a strong peak at 1730.8 cm⁻¹ for ester carbonyl stretching, along with bands from 1383.7 to 1053.9 cm⁻¹ related to C–O stretching. Alkyl –CH_3_ groups appear at 2984.4 and 2933.2 cm⁻¹, with corresponding symmetric and asymmetric bending vibrations at 1383.7 and 1457.9 cm⁻¹. A broad band at 3444.2 cm⁻¹ indicates terminal –OH groups^34,35^. PHB-diol (Fig. 1A_c_) exhibits characteristic peaks at 3438 cm⁻¹ (O–H), 2935 cm⁻¹ (C–H), 1720 cm⁻¹ (C = O), 1460 cm⁻¹ (C–C), and 1282–1122.4 cm⁻¹ (C–O), confirming successful transesterification^36^. CS (Fig. 1A_d_) displays –OH and C–H stretching at 3450 and 2918 cm⁻¹, respectively. Amide I and II bands are present at 1657 and 1581.6 cm⁻¹, while peaks at 1435 and 1385 cm⁻¹ correspond to C–H and O–H bending. Additional bands at 1159 cm⁻¹ (C–O–C bridge) and 1090–1038 cm⁻¹ (C–O stretching) are also observed^37,38^. The FTIR spectrum of PHHCS (Fig. 1A_e_) shows distinct bands at 1776.4, 1711.8, and 721.3 cm⁻¹, attributed to phthalimido carbonyl groups, tertiary amines, and aromatic ring vibrations, respectively, confirming successful phthaloylation of CS^39,40^.

The FTIR spectrum of PHB-co-PHHCS (Fig. 1A_f_) shows characteristic bands at 3452 cm⁻¹ (bonded –OH), 1638 cm⁻¹ (amide C = O stretching), and 1718 cm⁻¹ (aromatic phthalimido ring), confirming successful copolymer formation. In PHB-co-HCS (Fig. 1A_g_), the disappearance of phthalimide peaks at 1718 and 721 cm⁻¹ indicates effective deprotection^41^. A broad absorption at 3449 cm⁻¹ is assigned to overlapping N–H and hydrogen-bonded –OH groups. Peaks at 1440 cm⁻¹ (N–H bending of urethane) and 1641 cm⁻¹ (C = O stretching) confirm urethane formation, while the broad band at 1079 cm⁻¹ corresponds to C–N stretching collectively confirming the successful synthesis of PHB-co-HCS.

In the PHB-co-HCS/5%Co₃O₄ nanocomposites, the C = O peak shifted from 1641 to 1648 cm⁻¹, and the N–H band from 1440 to 1431 cm⁻¹. Additionally, Co–O stretching bands shifted from 676 to 575 cm⁻¹ to 666 and 577 cm⁻¹ respectively, indicating incorporation of Co₃O₄ into the polymer matrix. Similarly, in PHB-co-HCS/10%Co₃O₄, C = O and N–H bands appeared at 1647 and 1587 cm⁻¹, respectively, with Co–O signals at 667.3 and 579.5 cm⁻¹. These spectral shifts further confirm successful embedding of Co₃O₄ within the nanocomposite structure^42^. Comparable FTIR spectra were observed for PHB-co-LCS and its derivatives, including PHLCS, PHB-co-PHLCS, PHB-co-LCS/5%Co₃O₄, and PHB-Co-LCS/10%Co₃O₄, as illustrated in (Fig. S_1_).

The XRD pattern of Co₃O₄ nanoparticles (Fig. 1B_a_) displays distinct diffraction peaks at 2θ ≈ 18.04° (111), 31.5° (220), 36.73° (311), 39.59° (222), 44.65° (400), 59.28° (511), and 65.14° (440), characteristic of the spinel Co₃O₄ structure and consistent with JCPDS file no. 42-1467 ^43,44^. The average crystallite size was calculated using the Scherrer equation and estimated to be 18.36 nm.

Neat PHB (Fig. 1B_b_) exhibits peaks at 2θ ≈ 13.57° (020), 16.6° (110), 21.5° (101), 22° (111), 25.06° (121), 26.8° (040), and 36.34° (002), indicating its semi-crystalline nature^45^. Pure CS presents two broad peaks near 2θ ≈ 10° and 20°, corresponding to the (101) and (020) planes, respectively, reflective of low crystallinity and a partially amorphous structure^46^.

For PHB-co-HCS copolymers, the XRD pattern features new peaks at 2θ ≈ 9.3°, 30.64°, and 35°, suggesting the emergence of a new crystalline phase and confirming successful chemical coupling between PHB and CS. In PHB-co-HCS/10%Co₃O₄ nanocomposites, prominent Co₃O₄ diffraction peaks are observed at 2θ ≈ 31.48° (220), 37.05° (311), 44.8° (400), 59.4° (511), and 65.3° (440), along with polymeric peaks at 2θ ≈ 15.2°, 21.02°, and 23.58°. The high intensity of Co_3_O_4_ signals may mask some polymeric features, supporting successful nanoparticle incorporation into the copolymer matrix.

Similar diffraction patterns and phase confirmations were identified for PHB-co-LCS and PHB-co-LCS/10%Co₃O₄ nanocomposites (Fig. S_2_). The crystallite sizes determined in this study are consistent with previously reported values for chitosan-Co₃O₄ and FA-CS-Co₃O₄ nanocomposites, which were approximately 19.45 nm and 16.36 nm, respectively^47^.

**Fig. 2:**
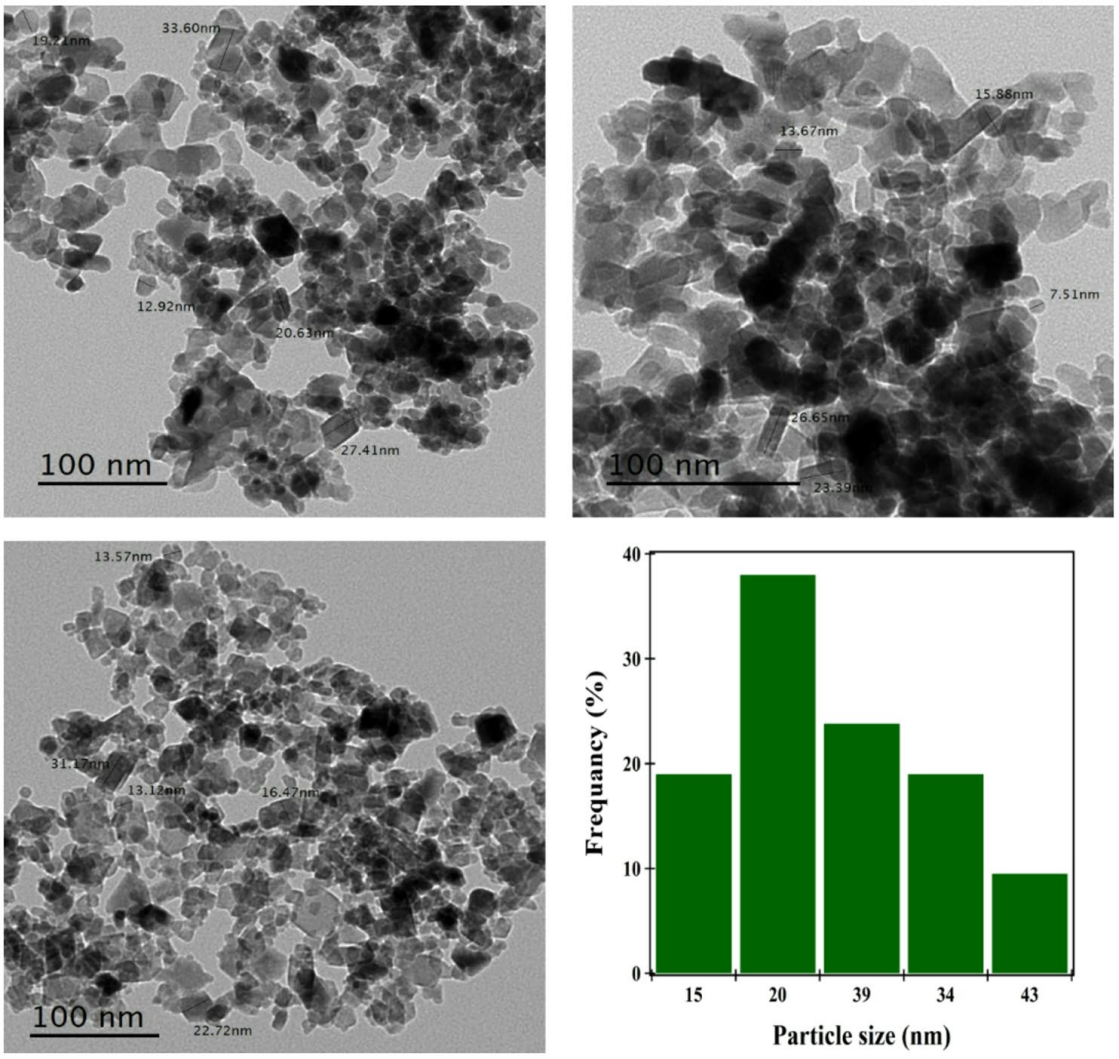
TEM micrograph of Co_3_O_4_ NPs, histogram of the particle size distribution.

**Fig. 3:**
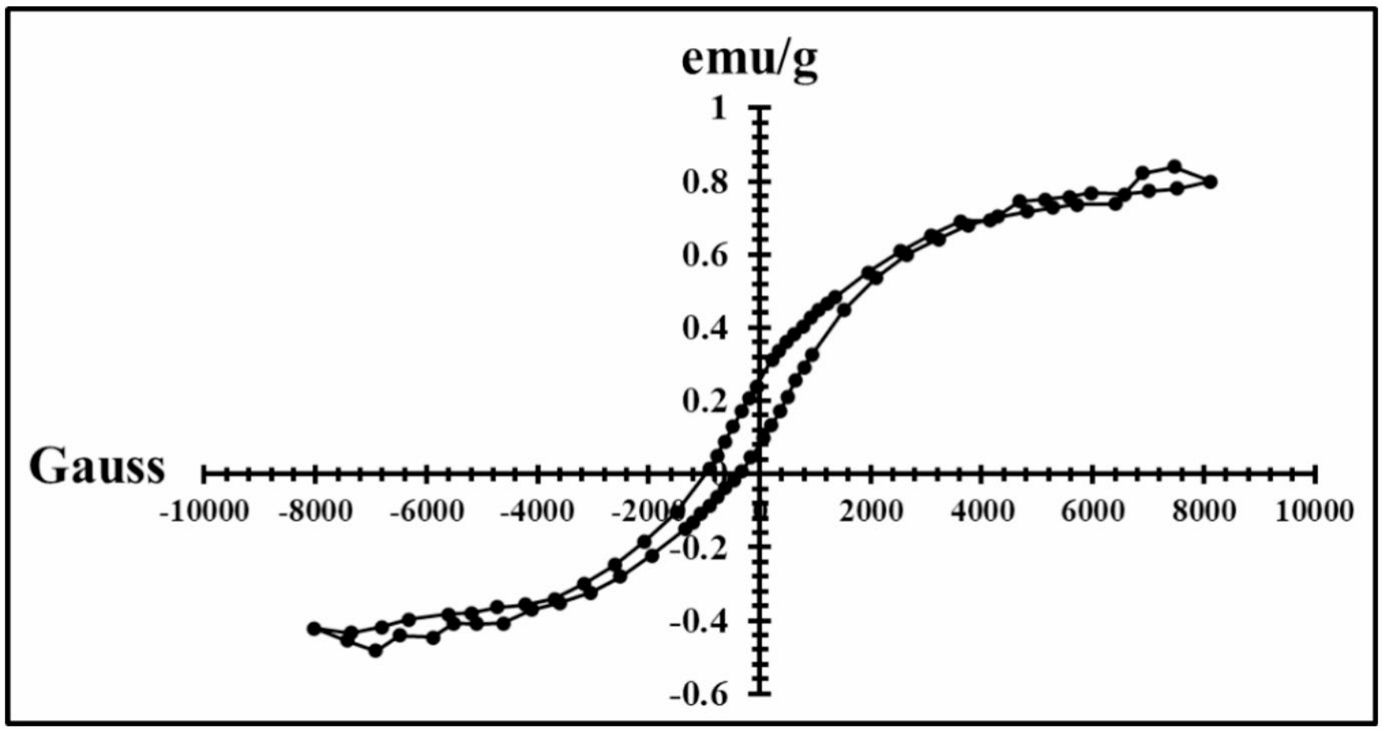
Magnetization vs. applied magnetic field for Co_3_O_4_ NPs.

The TEM image of the synthesized Co₃O₄ NPs (Fig. 2) reveals predominantly irregular morphologies with particle sizes ranging from 15 to 43 nm. These dimensions are consistent with previously reported HR-TEM studies, which documented Co₃O₄ nanoparticles with sizes between 19 and 41 nm ^48^. The observed nanoscale characteristics confirm the successful formation of well-dispersed nanoparticles suitable for NC integration. Isothermal magnetization (M–H) analysis of Co₃O₄ NPs (Fig. 3) yielded a saturation magnetization (Ms) of 0.8 emu g⁻¹, indicating weak ferromagnetic behavior. Compared to prior studies where Co₃O₄ samples exhibited lower Ms values of 0.23 emu g⁻¹ at 50 K and as low as 0.125 emu. g⁻¹ in other conditions^49^ these results highlight the influence of synthetic parameters such as temperature, solvent environment, and precursor selection on magnetic properties. It is worth noting that ferromagnetic behavior can be used in hyperthermia to generate heat by applying a magnetic field for cancer treatment.

**Fig. 4:**
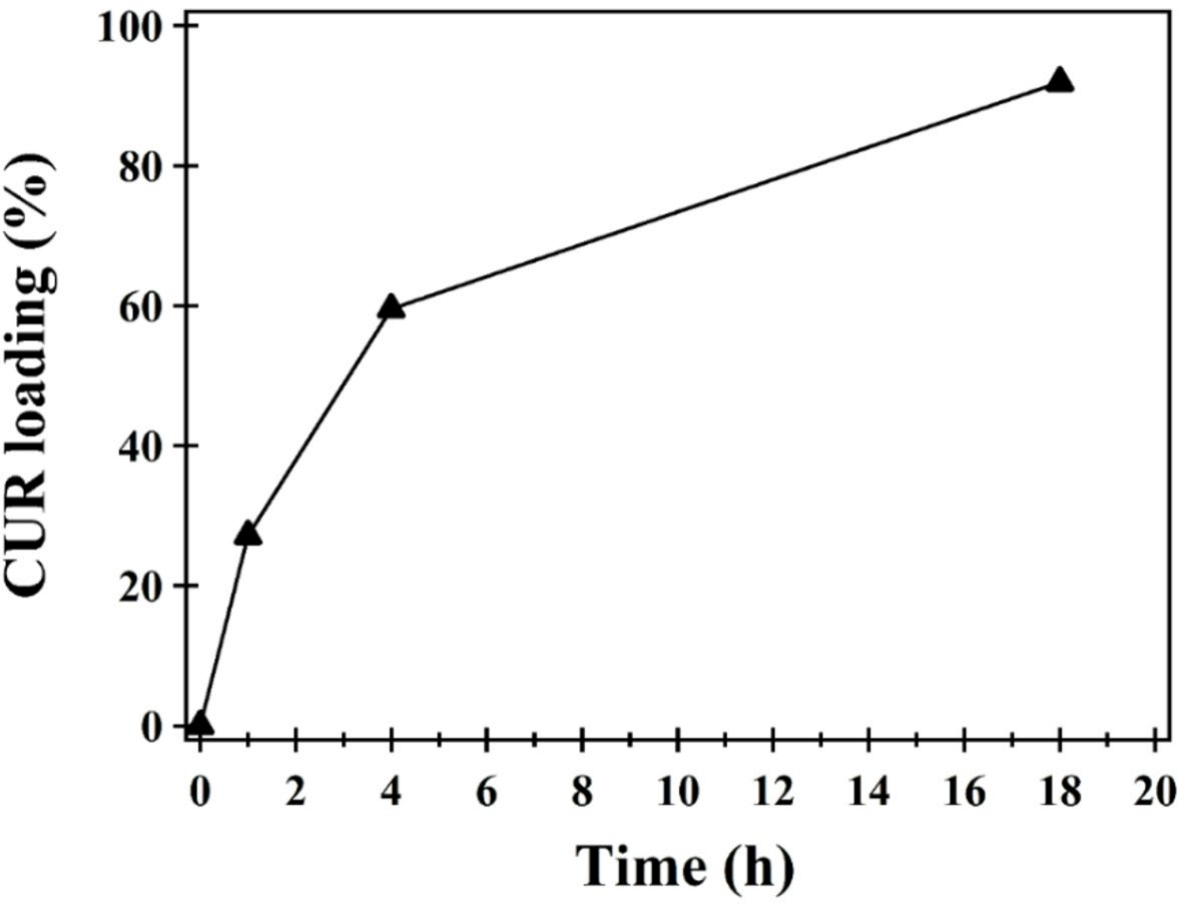
Loading (%) of CUR onto Co_3_O_4_ NPs.

The CUR loading efficiency onto Co_3_O_4_ NPs is depicted in (Fig. 4). A high loading capacity of **91.5%** was achieved, comparable to previously reported cobalt NP systems with FCC structures demonstrating ~ **89%** efficiency^50^. This high CUR loading is attributed to electrostatic interactions between the hydroxyl groups of CUR and the Co_3_O_4_ NPs surfaces, which support both magnetic behavior and targeted drug delivery capability^50^. It is worth to mentioning that loading capacity was affected by molecular weight^51^, and the concentration of polymer^52^.

**Fig. 5:**
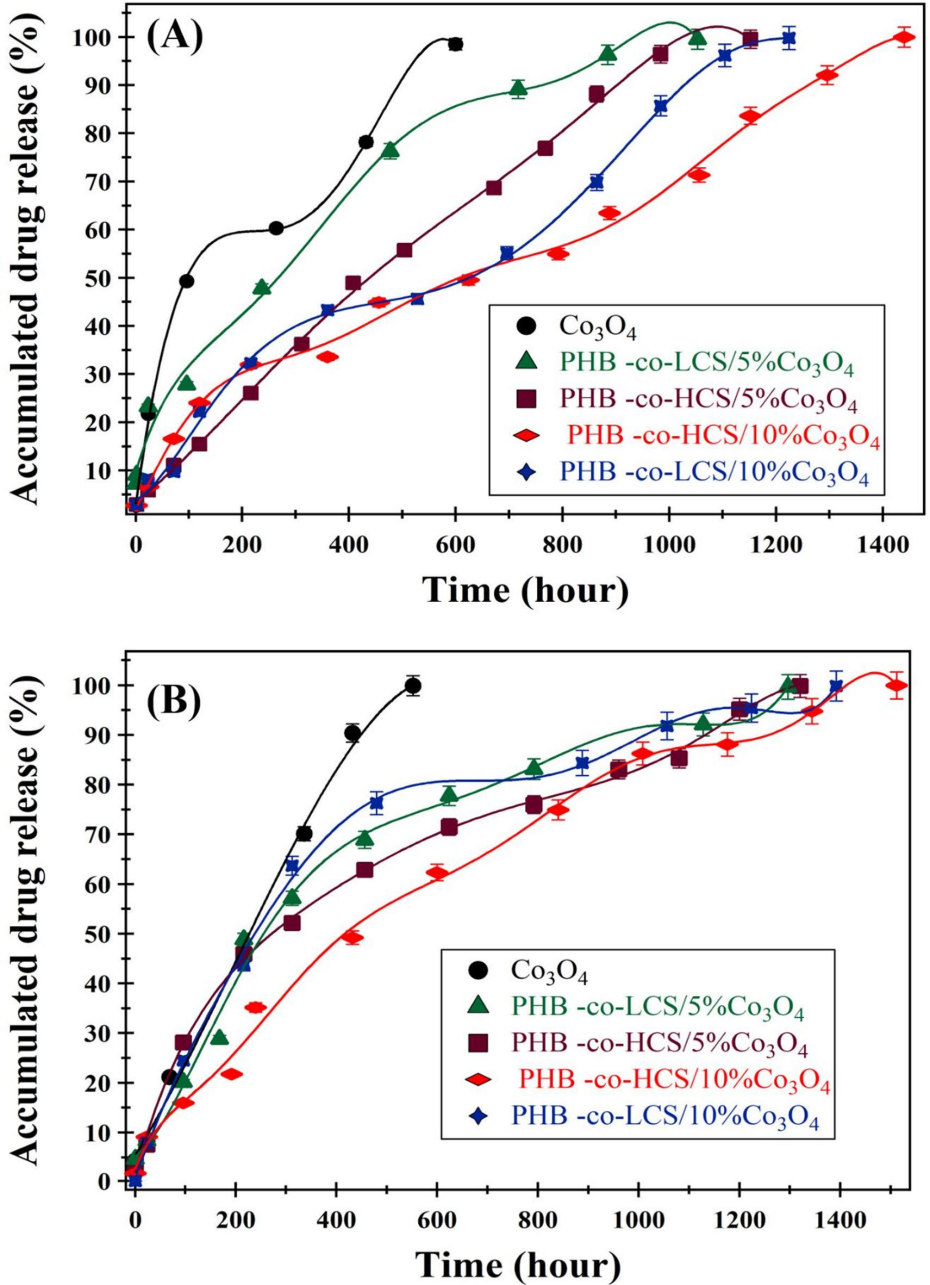
In vitro release of CUR loaded Co_3_O_4_, PHB-co-LCS/5%Co_3_O_4_, PHB-co-HCS/5%Co_3_O_4_, PHB-co-LCS/10%Co_3_O_4_ nanocomposites, and PHB-co-HCS/10%Co_3_O_4_ nanocomposites at pH 5.4 (**A**), and pH 7.4 (**B**).

The cumulative release profile of CUR from various nanocarriers Co₃O₄, PHB-co-HCS/5%Co₃O₄, PHB-co-HCS/10%Co₃O₄, PHB-co-LCS/5%Co₃O₄, and PHB-co-LCS/10%Co₃O₄ nanocomposites was examined at 37 °C under two pH conditions (5.4 and 7.4), simulating acidic and physiological environments. As shown in (Fig. 5), 50% CUR release was achieved within 94, 312, 240, 432, and 290 h for the respective systems at pH 5.4, and within 219, 504, 312, 624 h, and 696 h at pH 7.4. Complete CUR release from the Co₃O₄ nanocarrier occurred after 657 h at pH 5.4 and 552 h at pH 7.4. For PHB-based nanocomposites, 100% release at pH 7.4 was reached within 1296 h (PHB-co-LCS/5%Co₃O₄), 1320 h (PHB-co-HCS/5%Co₃O₄), 1392 h (PHB-co-LCS/10%Co₃O₄), and 1512 h (PHB-co-HCS/10%Co₃O₄). At pH 5.4, full release occurred within 1053, 1152, 1224, and 1440 h for the same respective formulations. These results confirm an accelerated release at acidic pH, which favors tumor-targeted delivery. The presence of Co₃O₄ NPs contributed to slower release, with higher NP content further delaying CUR diffusion. Conversely, the use of lower molecular weight chitosan increased the release rate due to enhanced solubility and reduced polymer-drug affinity. This behavior aligns with previous studies on PLGA systems, where higher molecular weights resulted in more sustained drug release^53^. Additionally, chitosan-coated Co₃O₄ NPs have shown enhanced release in acidic environments, facilitating cobalt ion diffusion into the medium^19^. In other publications, the rate of release was affected by molecular weight of CS^54^ in 5Fu@Cs, concentration of polyvinyl alcohol^47^ and the pH of the release medium^55^.

The drug release kinetics were analyzed using multiple mathematical models, including Zero-order, First-order, Higuchi, Hixson–Crowell, and Korsmeyer–Peppas equations [58,59]. For each model, the slope, regression coefficient (R^2^), release exponent (n), and rate constant (K) were calculated from the corresponding plots to elucidate the underlying release mechanisms, as summarized in (Table S_1_). At pH 5.3, both PHB-co-LCS/10%Co₃O₄ and PHB-co-HCS/10%Co₃O₄ nanocomposites followed Zero-order kinetics, indicating a constant release rate independent of drug concentration. In contrast, at pH 7.4, the release profiles for the same formulations were better described by the Higuchi model, suggesting diffusion-controlled release from the polymeric matrix. Moreover, analysis using the Korsmeyer–Peppas model revealed that the release mechanism in both media followed Fickian diffusion (*n* < 0.5), confirming that drug transport was governed by diffusion through the polymer matrix. Sustained release of metformin hydrochloride and sodium diclofenac from Gum ghatti-cl-poly(Acrylic acid)/ CoFe_2_O_4_)Gg-cl-poly(AA)/CoFe_2_O_4)_ hydrogels at pH 4 was following Fickian diffusion mechanism^56^.

**Fig. 6:**
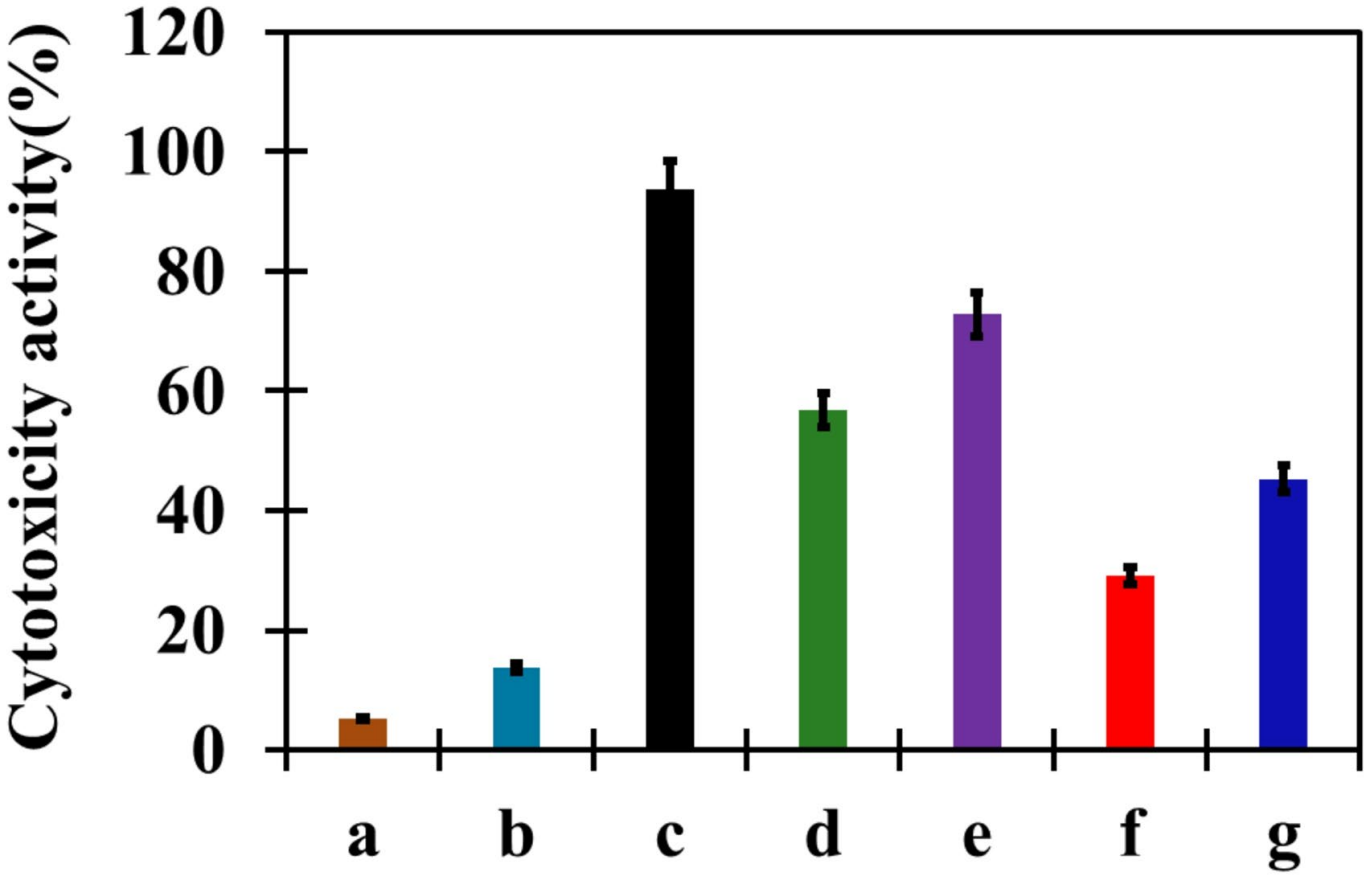
Inhibitory activity of (a) DOX, (b) CUR (c) CUR@Co_3_O_4_, (d) PHB-co-LCS /5%CUR@Co_3_O_4_, (e) PHB-co-HCS/5%CUR@Co_3_O_4_, (f) PHB-co-LCS/ 10%CUR@Co_3_O_4_, and (g) PHB-co-HCS/10%CUR@Co_3_O_4_ nanocomposites.

The cytotoxic effects of CUR@Co₃O₄, PHB-co-LCS/5%CUR@Co₃O₄, PHB-co-HCS/5%CUR@Co_3_O_4_, PHB-co-LCS/10%CUR@Co_3_O_4_, and PHB-co-HCS/10% CUR@Co_3_O_4_ nanocomposites were evaluated against HCT-116 colorectal cancer cells using the MTT assay, and IC_50_ values were determined (Fig. 6; Table 1). The calculated IC_50_ values were 96.69 µg/mL (CUR@Co_3_O_4_), 72.86 µg/mL (PHB-co-LCS/5%CUR@Co_3_O_4_), 56.77 µg/mL (PHB-co-HCS/5%CUR@Co_3_O_4_), 29.1 µg/mL (PHB-co-LCS/10%CUR@Co_3_O_4_), and 45.27 µg/mL (PHB-Co-HCS/10%CUR@Co_3_O_4_). Among these, PHB-co-LCS/10%CUR@Co_3_O_4_ nanocomposites exhibited the lowest IC_50_ value, indicating the highest anticancer efficacy.

Previous studies have reported that Co_3_O_4_ NPs combined with the proteasome inhibitor Carfilzomib (Cfz) promote accumulation of autophagy substrates, induce endoplasmic reticulum stress, and enhance protein ubiquitination-mechanisms that contribute to cancer inhibition^27^. Additionally, chitosan and its derivatives have demonstrated selective permeability to cancer cell membranes and exert anticancer effects through diverse mechanisms, including enzyme modulation, antiangiogenic activity, antioxidant defense, apoptosis induction, and immunomodulatory responses^57^. For comparison, poly(3-hydroxybutyrate)/chitosan-graft poly(acrylic acid) conjugated with sodium hyaluronate was previously reported to exhibit an IC₅₀ value of 11.7 µg/mL, with 67.88% apoptosis in Caco-2 cells^58^, emphasizing the importance of material design in enhancing therapeutic outcomes. Glucose-coated cobalt oxide NPs conjugated with Ellagic acid (Co₃O₄@Glu-Ellagic acid) selectively inhibited liver cancer cell proliferation by increasing ROS, inducing apoptosis, and causing cell cycle arrest^59^.

**Table 1:** In vitro cytotoxicity of from CUR, CUR@Co_3_O_4,_ PHB-co-HCS/5%CUR@Co_3_O_4,_ PHB-co-LCS/5%CUR@Co_3_O_4,_ PHB-co-LCS/10%CUR@Co_3_O_4_, and PHB-coHCS/10%CUR@Co_3_O_4_ nanocomposites on against HCT-116 cancer expressed as 50%.

No.	Compound	In vitro Cytotoxicity IC_50_ (µg/ml) HCT116
•	DOX	5.23 ± 0.3
1	CUR	10 µM.^60^
2	Sprague Dawley rats implanted with two 2-cm curcumin implants	60 ± 20 ng/g^6^
3	curcumin loaded selenium nanoparticles (Se-CurNPs)	13.5^61^
4	poly lactic acid-hyaluronic acid /Fe_3_O_4_/Curcumin NPs	52.26 ± 4.3^62^
5	curcumin Emulsomes	19.69 ± 3.27 µM^63^
6	CUR	13.72 ± 1.1
7	CUR@Co_3_O_4_	93.69 ± 4.9
8	PHB -co-HCS/5%CUR@Co_3_O_4_	56.77 ± 3.1
9	PHB -co-LCS/5%CUR@Co_3_O_4_	72.86 ± 3.5
10	PHB -co-LCS/10%CUR@Co_3_O_4_	29.10 ± 2.0
11	PHB -co-HCS/10%CUR@Co_3_O_4_	45.27 ± 2.7

## Conclusion

This study developed a PHB-co-CS/Co_3_O_4_ nanocomposite for the loading and controlled release of CUR in colorectal cancer therapy. The copolymer was synthesized via a coupling reaction between the terminal hydroxyl groups of PHB-diol and the isocyanate functionalized chitosan, using chitosan of varying molecular weights and varying the concentrations of Co_3_O_4_ NPs. FTIR analysis confirmed the successful synthesis of PHB-co-HCS and the effective incorporation of Co_3_O_4_ into the polymer matrix. In addition, XRD patterns revealed the emergence of a new crystalline phase, validating the chemical coupling between PHB and chitosan and further supporting the successful incorporation of Co_3_O_4_ NPs into the copolymer structure. TEM analysis confirmed irregularly shaped Co_3_O_4_ NPs with sizes ranging from 14 to 43 nm and a measured saturation magnetization of 0.8 emu g⁻¹. CUR was effectively loaded onto Co₃O₄ and dispersed within the PHB-co-CS matrix. In-vitro release studies were conducted in buffer solutions at pH 5.4 and 7.4, showing enhanced release in acidic conditions, which supports tumor-specific targeting. Formulations with lower molecular weight chitosan released curcumin more rapidly. Nanocomposites with higher contents of Co₃O₄ revealed slower release due to delayed CUR diffusion. Drug release kinetics followed a Zero-order model at pH 5.3 and a Higuchi model at pH 7.4, indicating a transition from concentration-independent to diffusion-controlled release mechanisms. Among all formulations, PHB-co-LCS/10% CUR@Co₃O₄ demonstrated the most potent cytotoxic effect with an IC₅₀ of 29.1 µg/mL against HCT-116 colorectal cancer cells, exhibiting the lowest IC₅₀ value.

## Supplementary Information

Below is the link to the electronic supplementary material.

Supplementary Material 1

## Data Availability

All datasets produced or examined throughout this investigation are fully presented within the published manuscript.
