# Supplementary material for: Curcumin delivery system based on biodegradable polyhydroxybuterate Chitosan copolymer and Cobalt oxide nanoparticles against colorectal cancer
Source: Sci Rep. 2026 Mar 9;16:8722. doi: 10.1038/s41598-025-34587-y (PMC12979646; doi:10.1038/s41598-025-34587-y)
Supplement: Supplementary file 1 — Supplementary Material 1 [file 41598_2025_34587_MOESM1_ESM.docx]

**Controlled Release of Curcumin from Polyhydroxybutyrate-Co-Chitosan/Cobalt Oxide Nanocomposites: A Biodegradable Platform for Colorectal Cancer Treatment**

Nehal Salahuddin*, Mohamed Gaber, Maie Mousa, Mona Elfiky*

Chemistry Department, Faculty of Science, Tanta University, 31527, Egypt.

*Tel.: +0115429713; fax: +91 1332 273560*

**Corresponding author e-mail:* [*Elfiky_mona@science.tanta.eu.eg*](mailto:Elfiky_mona@science.tanta.eu.eg) *&* [nehal.attaf@science.tanta.edu.eg](mailto:nehal.attaf@science.tanta.edu.eg)

The FTIR spectrum of Co_3_O_4_, PHB, PHB-diol, CS, PHB-Co-PHLCS copolymers, PHB-Co-LCSCP, PHB-Co-LCS-5%Co_3_O_4,_ and PHB-Co-LCS-10%Co_3_O_4_ were shown in **(Fig. S_1_)**. The spectrum LPHCS (**Fig. S_1_d)** shows bands at 1776.35, 1711.8, and 720.7 cm^−1^, assigned to the carbonyl, the tertiary amino, and the aromatic ring of the phthalimido groups, respectively[^1^](#_ENREF_1), confirming formation of LPHCS. The spectra of PHB-Co-LPHCS (**Fig. S_1_e),** shows that the band at 3451 cm^−1^ may be attributed to bonded -OH-groups. The band at 1638.23 cm^-1^ corresponds to C=O stretching modes. The band at 1776.35 cm^-1^ corresponds to the aromatic ring of the phthalimido group. FTIR spectra of PHB-Co-LCS **(Fig. S_1_f),** revealed the disappearance of the characteristic bands of phthalimide groups at 1776.35, and 720.8 cm^-1^, indicating that phthalimide group of PHB-Co-LPHCS was deprotected [^2^](#_ENREF_3). In the spectra of PHB-Co-LCS the band at 3447 cm^-1^ may be attributed to bonded -NH-groups that are overlapped with hydrogen-bonded hydroxyl groups with CS, and absorption band becomes more distinct. The appearance of band centered at 1443 cm^−1^ is attributed to the bending vibration of NH of the urethane moiety. The band at 1639 cm^-1^ corresponds to C=O stretching modes. The appearance of a broad absorption band at 1079 cm^-1^ is attributed to the stretching vibration of nanocomposite. These results together confirm the formation of copolymer PHB-Co-LCS (**Fig. S_1_f**). In the spectrum of PHB-Co-LCS/5%Co_3_O_4_ nanocomposite, the band characteristic to the C= O group was shifted from 1641 to 1645 cm^-1^, and the band belonging to N–H unit in urethane linkage was shifted from 1440 to 1443cm^-1^. Also, the band assigned to the stretching of Co_3_O_4_ was shifted from 670 to 668 cm^-1^ and from 575 to 579 cm^-1^. This result confirms the inclusion Co_3_O_4_ in the nanocomposite. In the spectrum PHB-Co-LCS/10%Co_3_O_4_ nanocomposite show that the peak characteristic to the C= O group was shifted from 1639 to 1646 cm^-1^ and 1589 cm ^-1^ belonging to N–H unit in urethane linkage was observed[^3^](#_ENREF_4). In addition, the band assigned to the stretching of Co-O bonds appears at at667.25 and 577.58 cm^-1^ has appeared in the nanocomposites. This result confirms the inclusion of Co_3_O_4_ in the nanocomposites.


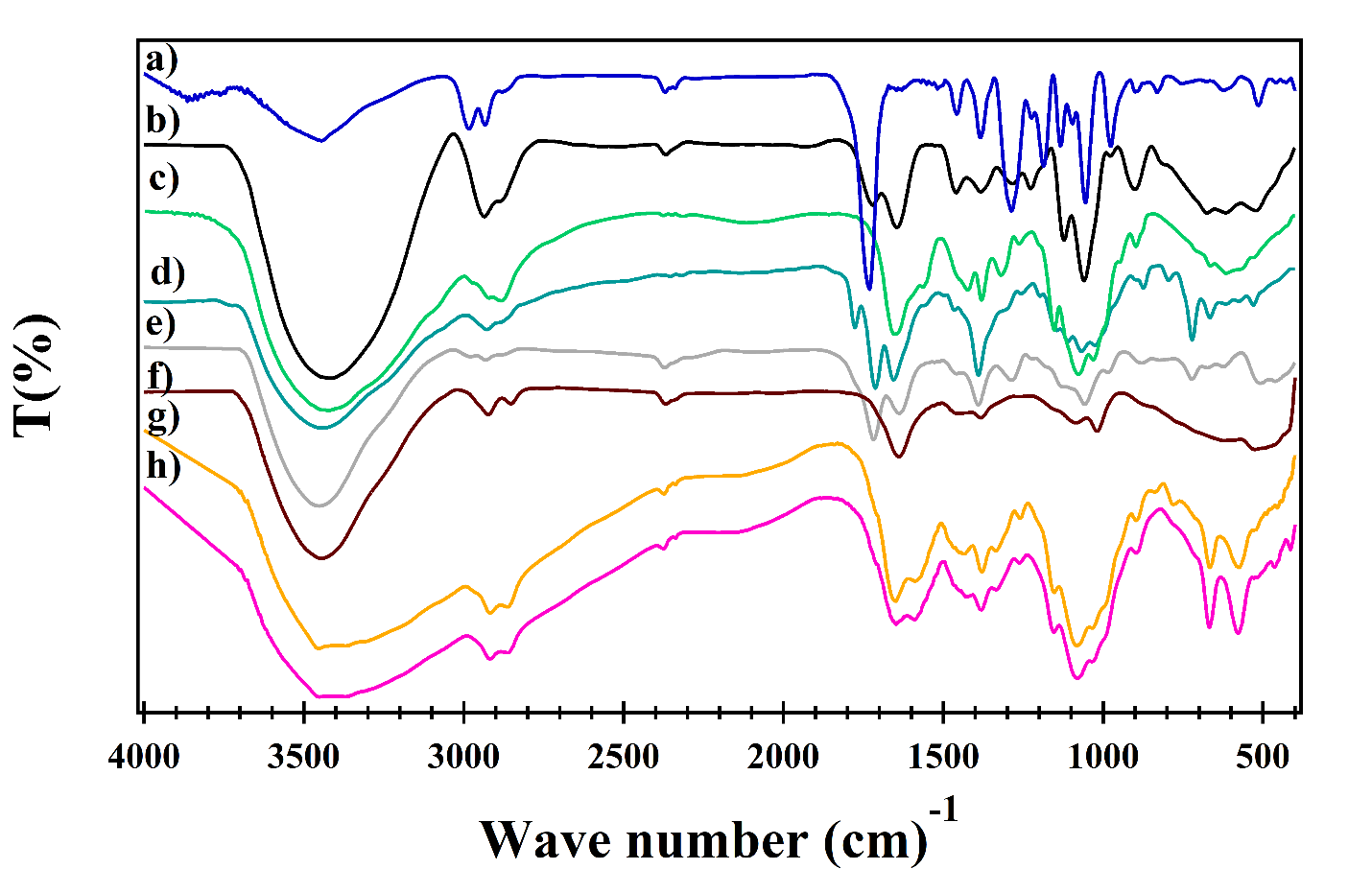


**Figure S_1_.** FTIR spectra of a) PHB, b) PHB-diol, c) CS, d) PHLCS, e) PHB-Co-PHLCS, f) PHB-Co-LCS CP, g) PHB-Co-LCS/5%Co_3_O_4_ nanocomposite, and h) PHB-Co-LCS/ 10%Co_3_O_4_ nanocomposite.

The XRD pattern of PHB-Co-LCS reveals the existence of new sharp crystalline band at 2θ ≈ 9.28°, 30.47° and 34.98° confirming the formation of a new crystalline structure, and successful coupling reaction between the PHB, and CS polymers had occurred. The XRD pattern of PHB-Co-LCS/10%Co_3_O_4_ nanocomposite shows that the characteristic sharp bands of Co_3_O_4_ at 2θ ≈ 31.326°, 36.98°, 44.89°, 59.46°, and 65.32° with three bands at 2θ ≈ 15.6°, 21.058°, and 23.945° may be characteristic to PHB-Co-HCS. It is worth noting that the presence of sharp bands of Co_3_O_4_ may lead to the difficulty to observe the bands characteristic to PHB-Co-CS, which confirms the incorporation of Co_3_O_4_ into the PHB-Co-CS copolymer matrices.


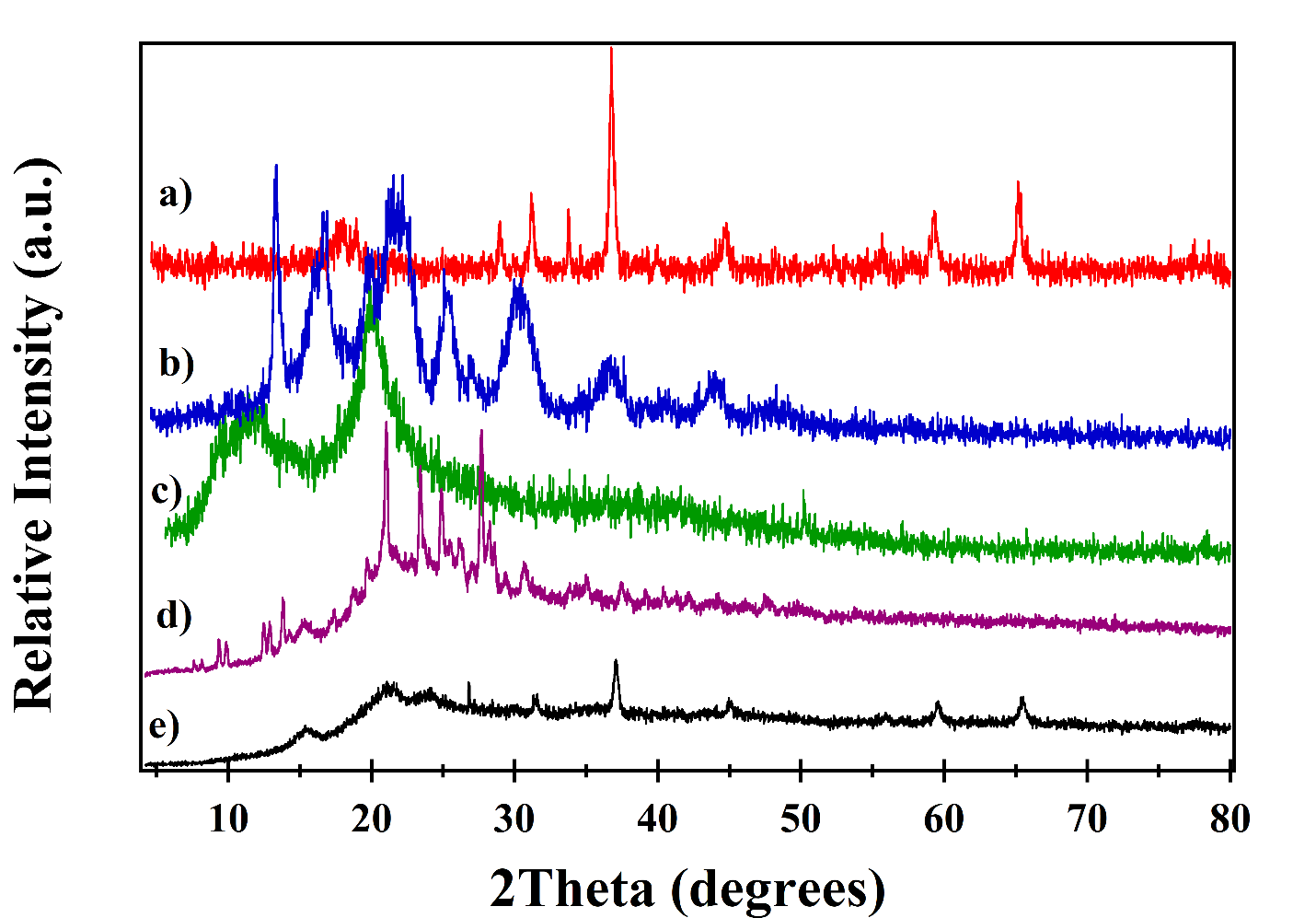


**Figure S_2_.** XRD pattern a) Co_3_O_4_ NPs, b) PHB, c) LCS, d) PHB-Co-LCS copolymer and e) PHB-Co-LCS/10% Co_3_O_4_ nanocomposite.

| **Compound** | **pH** | | **Zero-order** | | **First-order** | | **Higuchi** | | **Hixson-Crowell** | | **Korsemeyer-Peppas** | | |
| --- | --- | --- | --- | --- | --- | --- | --- | --- | --- | --- | --- | --- | --- |
|  |  |  | **K_1_** | **R^2^** | **K_2_** | **R^2^** | **K_3_** | **R^2^** | **K_4_** | **R^2^** | **K_5_** | **R^2^** | **n** |
| **CUR** | | | | | | | | | | | | | |
| **PHB-Co-HCS/10%Co_3_O_4_** | | **5.3** | **0.0614** | **0.977** | **-0.0013** | **0.730** | **2.523** | **0.960** | **-0.0021** | **0.833** | **-1.82** | **0.983** | **0.55** |
| **PHB-Co-LCS/10%Co_3_O_4_** | |  | **0.0754** | **0.984** | **-0.0019** | **0.753** | **3.005** | **0.961** | **-0.003** | **0.898** | **-1.85** | **0.965** | **0.58** |
| **PHB-Co-HCS/10%Co_3_O_4_** | | **7.4** | **0.061** | **0.954** | **-0.0019** | **0.751** | **2.771** | **0.989** | **-0.0026** | **0.951** | **-1.82** | **0.987** | **0.57** |
| **PHB-Co-LCS/10%Co_3_O_4_** | |  | **0.0583** | **0.933** | **-0.0019** | **0.868** | 2.689 | **0.97** | **-0.0027** | **0.967** | **-2.33** | **0.957** | **0.77** |

**Table S_1_.** Controlled release kinetics of CUR from PHB-Co-LCS/10%Co_3_O_4_ NC, and PHB-Co-HCS/10%Co_3_O_4_ NC at different pH media

**References**

(1) (a) Zhou, W.; Wang, Y.; Jian, J.; Song, S., Self-aggregated nanoparticles based on amphiphilic poly (lactic acid)-grafted-chitosan copolymer for ocular delivery of amphotericin B. *International journal of nanomedicine* **2013,** *8*, 3715; (b) Liu, L.; Shi, A.; Guo, S.; Chen, S.; Li, J., Preparation of chitosan-g-polylactide graft copolymers via self-catalysis of phthaloylchitosan and their complexation with DNA. *Reactive and Functional Polymers* **2010,** *70* (5), 301-305.

(2) Chen, W.; Huang, D.; Hu, Z.; Zhuang, Y.; Lu, S. In *Preparation and characterization of 6-O-caffeic acid chitosan*, Journal of Physics: Conference Series, IOP Publishing: 2021; p 012029.

(3) Pan, J.; Li, G.; Chen, Z.; Chen, X.; Zhu, W.; Xu, K., Alternative block polyurethanes based on poly (3-hydroxybutyrate-co-4-hydroxybutyrate) and poly (ethylene glycol). *Biomaterials* **2009,** *30* (16), 2975-2984.
